# Assessment of quality, reliability, and interactive characteristics of age-related macular degeneration short videos on TikTok: a cross-sectional study

**DOI:** 10.1038/s41598-026-44509-1

**Published:** 2026-04-30

**Authors:** Li Zhong, Siyan Liu, Wei Lu, Qi Wu, Yuxin Lv, Shuangshuang Yi, Xuan Wang, Zhengzheng Wu, Jing Yan

**Affiliations:** 1 China Academy of Chinese Medical Sciences Data Center, No. 16, Nanxiaojie, Dongzhimennei Ave, Dongcheng District, Beijing, 100700 China; 2 China Academy of Chinese Medical Sciences Eye Hospital, No. 33, Lugu Street, Shijingshan District, Beijing, 100040 China; 3 Anshan Hospital of Traditional Chinese Medicine, Anshan, Liaoning China; 4 Yanqing Hospital of Beijing Chinese Medicine Hospital, Beijing, China

**Keywords:** Age-related macular degeneration, TikTok, Social media, Video quality, Health education, Diseases, Health care, Mathematics and computing, Medical research

## Abstract

Age-related macular degeneration (AMD) is a leading cause of blindness in the elderly. Short-video platforms like TikTok are increasingly important sources of health information, yet concerns persist regarding content quality and reliability. To systematically evaluate the quality, reliability, and user engagement characteristics of AMD-related videos on TikTok. We systematically searched TikTok for AMD-related videos. Quality and reliability were assessed using JAMA benchmark, modified DISCERN, Global Quality Scale (GQS), and Patient Education Materials Assessment Tool (PEMAT). Entropy weight method and cluster analysis were applied to engagement data. Among 145 videos, overall quality was poor. Median scores were 1 for JAMA, 2 for mDISCERN, and 3 for GQS. Understandability was limited (median PEMAT-U: 43%), while actionability was moderate (median PEMAT-A: 60%). High-quality videos were characterized by creation by Western medicine ophthalmologists, inclusion of prognostic information, and monologue narration; physician title showed no association with quality. “Saves” carried the highest engagement weight (35.71%). "Self-test and screening" themes achieved the highest engagement rate (62.5%). Media accounts attained the highest PEMAT-U scores and interaction metrics. Video format and presenter attire showed no significant impact on quality or engagement. AMD-related health information on TikTok is generally poor quality. Information quality and user engagement are driven by distinct factors, highlighting the need for targeted strategies to improve content accuracy and understandability.

**Supplementary Information:**

The online version contains supplementary material available at 10.1038/s41598-026-44509-1.

## Introduction

Age-related macular degeneration (AMD) is a major cause of low vision and even blindness in the elderly population^1^. Globally, the prevalence of AMD is approximately 8.69%, and the number of AMD patients is projected to reach 288 million by 2040^2,3^. Meanwhile, AMD is the leading cause of blindness in people over 55 years old in Western countries^4^. In China, the prevalence of AMD in people over 70 years old is 20.2%^3^. With the intensification of population aging, the decline in quality of life and socioeconomic burden imposed by AMD will further escalate. Notably, in addition to uncontrollable factors such as age, race, and genetics, the early occurrence and development of AMD are also closely related to controllable factors such as smoking, diet, and blood lipid control. There is ample evidence that lifestyle changes such as smoking cessation, weight control, management of chronic diseases, and dietary modifications can reduce the risk of AMD onset and delay disease progression^5–7^. For advanced AMD, Anti-Vascular Endothelial Growth Factor (Anti-VEGF) therapy is the first-line treatment recommended by current guidelines, and its efficacy is highly dependent on patient treatment adherence^8^. AMD patients need to strictly follow medical advice to complete the treatment cycle and attend follow-up appointments on time, avoiding disease recurrence due to treatment discontinuation or delayed visits, which increases the difficulty and economic burden of subsequent treatment. Therefore, in the comprehensive management of AMD, on the one hand, we can reduce the risk of disease occurrence by popularizing knowledge on risk factor prevention and control; on the other hand, it is necessary to emphasize the necessity of standardized anti-VEGF therapy to patients, fully explain the importance of compliance, and help them establish awareness of standardized treatment. Only in this way can we comprehensively reduce the health damage and socioeconomic pressure caused by AMD.

As an emerging media form, short videos have gained rapid popularity worldwide in recent years, greatly changing the way people access information^9^. Short video platforms represented by TikTok have attracted a large number of users with their unique algorithm recommendation mechanisms and user-friendly creation tools^10^. According to the 51st Statistical Report on China’s Internet Development published by the China Internet Network Information Center (CNNIC), as of December 2022, the usage rate of short video users in China reached 94.8%, with the number of users reaching 1.012 billion. With the expansion of user scale, TikTok has not only become an important platform for social entertainment but also increasingly a key channel for the dissemination of various types of information, including healthcare and health education content.

However, the quality of health information on short video platforms such as TikTok varies greatly, which has attracted widespread attention. Studies have shown that a large number of health education content videos have problems such as incomplete information, lack of evidence, or misleading content, which may affect the public’s correct understanding of diseases and further interfere with their medical decision-making and treatment compliance^11–13^. For AMD, early intervention and standardized treatment are crucial to delaying vision loss, while erroneous or one-sided information may lead patients to delay diagnosis and treatment, blindly seek non-mainstream therapies, or even discontinue necessary anti-VEGF treatment.

Currently, there is a lack of systematic evaluation of the quality and communication effectiveness of AMD-related videos on TikTok. Therefore, this study aims to comprehensively evaluate the information quality, reliability, and interactive characteristics of AMD-related short videos on TikTok. The study employed the Journal of the American Medical Association (JAMA) benchmark to assess the accuracy and credibility of the videos, the modified DISCERN scale (mDISCERN) to evaluate reliability and objectivity, and the Global Quality Scale (GQS) to assess overall content quality. Additionally, the Patient Education Materials Assessment Tool (PEMAT) was used to evaluate understandability and actionability. These assessment tools have been widely applied and validated in prior research evaluating the quality of health information on social media^14–16^. Among them, Charnock originated the DISCERN tool in 1999 for evaluating textual health information^17^. However, this tool is not applicable for assessing the information quality of video materials^18^. In 2012, Singh.AG adapted Charnock’s DISCERN tool and created the modified DISCERN tool to evaluate the reliability of video materials^19^. Since then, this modified version has been widely used. Simultaneously, this study collects data on video attributes, publisher information, and user interaction metrics such as likes, comments, saves, and shares, analyzing their association with quality scores. In addition, this study further determined the weights of interaction indicators through the entropy weight method and conducted cluster analysis on this basis to reveal differences in content quality, creator background, information dimensions, and presentation forms between high-interaction and low-interaction videos.

Through the above analysis, this study hopes to provide a solid basis for improving the quality of AMD health education content, facilitating the dissemination and practical application of high-quality health knowledge.

## Materials and methods

### Ethical considerations

This study did not use any clinical data, human samples, or experimental animals. All information was obtained from publicly released TikTok short videos, and the data did not involve personal privacy issues. In addition, this study did not involve any user interaction, so ethical review was not required.

### Search strategy and data collection

On September 21, 2025, this study searched TikTok using the keyword "Age-Related Macular Degeneration", screening the top 200 videos by comprehensive ranking. To minimize potential bias from personalized recommendations,we registered and logged into a new TikTok account to conduct the search.

**Fig. 1:**
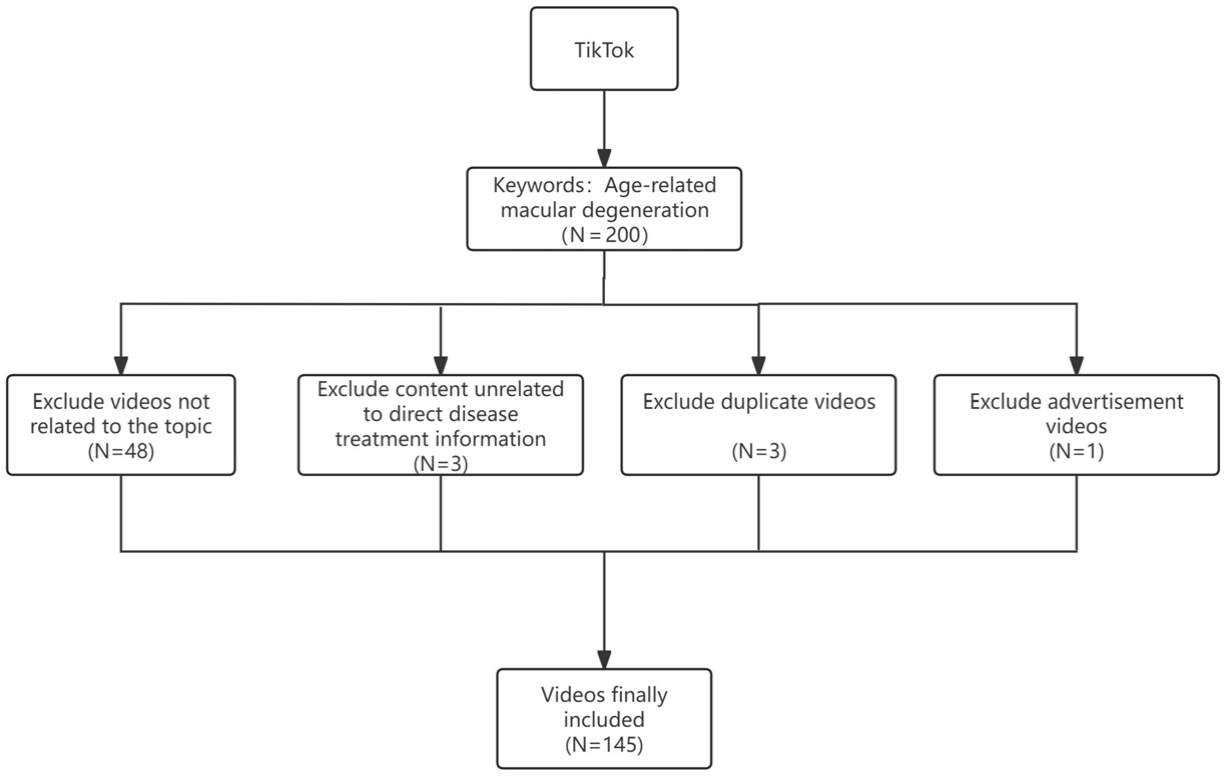
Video selection flowchart.

Exclusion criteria included (Fig. 1):

- Videos unrelated to the target keyword;
- Videos unrelated to disease health education content, such as issues related to medical insurance reimbursement for AMD;
- Duplicate videos; d. Commercial advertisements.

Following video inclusion, two researchers systematically extracted and standardized the video attribute indicators. Video format was categorized as “monologue narration” or “dialogue” based on whether the presenter engaged in direct communication with others. Treatment mention was defined as the video explicitly referring to any intervention for AMD. Creator professional titles and backgrounds were verified and annotated based on their TikTok account authentication information and official hospital website information. Visual presentation was recorded according to whether the presenter wore a white coat. All user interaction metrics (likes, comments, saves, shares) and basic attributes (video duration, upload time) were collected directly from the platform. All classifications were independently performed by two researchers, with any discrepancies resolved through discussion to reach consensus.

On the same day, the following data were systematically extracted and recorded in Microsoft Excel for subsequent analysis: basic video attributes (video duration, release time, media type); video content characteristics (presentation format, on-screen attire, mention of treatment or prognosis); user interaction metrics (number of likes, saves, comments, shares); and uploader information (professional title, number of followers, platform verification status). Video sources were categorized as Western Medicine Ophthalmologists, Traditional Chinese Medicine Ophthalmologists, Non-Ophthalmology Physicians, and News Media. Professional accounts were verified through platform details and hospital websites.

### Quality assessment

Two researchers independently conducted blind evaluations of video content using four validated tools: JAMA Benchmarks, mDISCERN scale, GQS, and PEMAT. These tools jointly form a comprehensive evaluation system from the perspectives of information credibility, objectivity, content quality, and educational effect.

The JAMA benchmark assesses accuracy and credibility from four dimensions: authorship, attribution of sources, disclosure of dates, and conflict of interest disclosure. Each criterion met scores 1 point, total score range 0 to 4, with higher scores indicating stronger information reliability.

The mDISCERN scale mainly evaluates the reliability and objectivity of health information, covering five dimensions: clarity of expression, reliability of sources, objectivity of content, extensiveness of resources, and explanation of uncertainties. Scoring uses 1–5 points, with higher total scores indicating more comprehensive, objective, and valuable information.

The Global Quality Scale (GQS) evaluates information quality overall, including completeness of information, clarity of structure, and suitability for patients. The scoring range is 1–5, with high scores indicating well-structured, easily understandable content with high educational value.

The Patient Education Materials Assessment Tool (PEMAT) is designed for audiovisual materials, assessing their understandability (PEMAT-U), such as language simplicity and structural clarity; and actionability (PEMAT-A), such as the specificity and feasibility of action recommendations. Scores for each are expressed as percentages, with higher percentages indicating materials that are easier for patients to understand and apply.

To ensure methodological rigor, the study employed an independent assessment by two researchers, who were blinded to each other’s scores during the initial evaluation stage. In case of disagreement, a third researcher was invited to participate in discussion to reach a consensus.

### Statistical analysis

This study used IBM SPSS Statistics for Mac, Version 26.0 for statistical analysis. For continuous variables that did not meet the assumption of normal distribution, the Wilcoxon rank-sum test was used for inter-group comparisons; continuous variables conforming to normal distribution were analyzed using independent samples t-test. Spearman correlation analysis was used to explore the relationships between variables. To explore the natural grouping characteristics of the sample on core research variables, cluster analysis was further employed, K-means cluster analysis was adopted for this purpose, and the optimal number of clusters (k = 2) was objectively determined based on the Silhouette Coefficient, a key index for evaluating cluster validity. A Silhouette Coefficient greater than 0.30 indicated that the two-cluster solution achieved the most distinct separation of the dataset (Table 2). For continuous variables not conforming to normal distribution, median (Q1, Q3) is reported; for continuous variables conforming to normal distribution, mean ± standard deviation is reported; categorical variables are reported as frequency and percentage. *P* < 0.05 was considered statistically significant. Additionally, this study used the entropy weight method to determine the objective weights of each interaction indicator (likes, comments, saves, shares). First, the original data were normalized, then the information entropy and information utility value of each indicator were calculated, and finally their weight coefficients were derived. All calculations were performed in the R language environment (version 4.3.1) using custom scripts.

## Statistical analysis results

### Descriptive statistical results and quality-engagement relationship

A total of 145 health education videos related to age-related macular degeneration (AMD) were included in this study. Overall information quality scores were generally low: the median JAMA score was 1.00 (Q1 = 0.0, Q3 = 3.0), the median mDISCERN score was 2.00 (Q1 = 0.0, Q3 = 4.0), and the median GQS score was 3.00 (Q1 = 0.0, Q3 = 5.0). The PEMAT showed that the median PEMAT-U (understandability) score was 0.429 (Q1 = 0.167, Q3 = 0.667), and the median PEMAT-A (actionability) score was 0.600 (Q1 = 0.0, Q3 = 1.0) (Table 1). Detailed frequency distributions of quality score indicators showed that JAMA scores were concentrated at 1 point (frequency > 100), mDISCERN scores were mainly at 2 points (frequency approximately 125), and GQS scores were concentrated at 3 points (frequency > 130) (Supplementary Fig. 1). User interaction indicators showed a highly skewed distribution: the median number of likes was 180.00 (range 0–83,584), comments was 8.00 (range 0–6451), saves was 55.00 (range 0–32,579), and shares was 33.00 (range 0–29,278) (Table 1).

**Table 1:** Descriptive statistics of core indicators for age-related macular degeneration short videos (n = 145).

Indicator	Average (SD)	Median (range)
JAMA score	1.083 ± 0.323	1 (0–3)
mDISCERN score	1.897 ± 0.621	2 (0–4)
GQS score	2.772 ± 0.926	3 (0–5)
PEMAT-U	0.394 ± 0.177	0.429 (0.167–0.667)
PEMAT-A	0.599 ± 0.135	0.6 (0–1)
Video length	1.315 ± 0.466	1 (0–2)
Likes	576.441 ± 1047.007	180 (0–83,584)
Comments	44.490 ± 844.490	8 (0–6451)
Saves	224.683 ± 824.683	55 (0–32,579)
Shares	201.917 ± 426.643	33 (0–29,278)
Number of followers	17.924 ± 6.635	2.9 (0.1–58.5)
Weighted sum of interactions	304.151 ± 503.008	104.25 (37.845–263.89)

PEMAT-U = Patient Education Materials Assessment Tool for understandability; PEMAT-A = Patient Education Materials Assessment Tool for actionability; GQS = Global Quality Scale.

Correlation analysis revealed that interaction indicators were significantly positively correlated with some quality indicators: likes were correlated with PEMAT-U and PEMAT-A; comments were correlated with mDISCERN score, GQS score, PEMAT-U, and PEMAT-A; saves were correlated with mDISCERN score, GQS score, PEMAT-U, and PEMAT-A; shares were correlated with mDISCERN score, GQS score, PEMAT-U, and PEMAT-A. The number of followers was also significantly positively correlated with JAMA score, PEMAT-U, and all interaction indicators. Quality score indicators were generally significantly positively correlated with each other: JAMA score was significantly correlated with mDISCERN score, GQS score, and PEMAT-A; mDISCERN score was significantly correlated with GQS score, PEMAT-U, and PEMAT-A; GQS score was significantly correlated with PEMAT-U and PEMAT-A; PEMAT-U and PEMAT-A were also significantly positively correlated. Video duration was significantly negatively correlated with JAMA score, GQS score, and PEMAT-A, but showed no significant correlation with any interaction indicators (Supplementary Fig. 3).

### Factors influencing user engagement

**Fig. 2:**
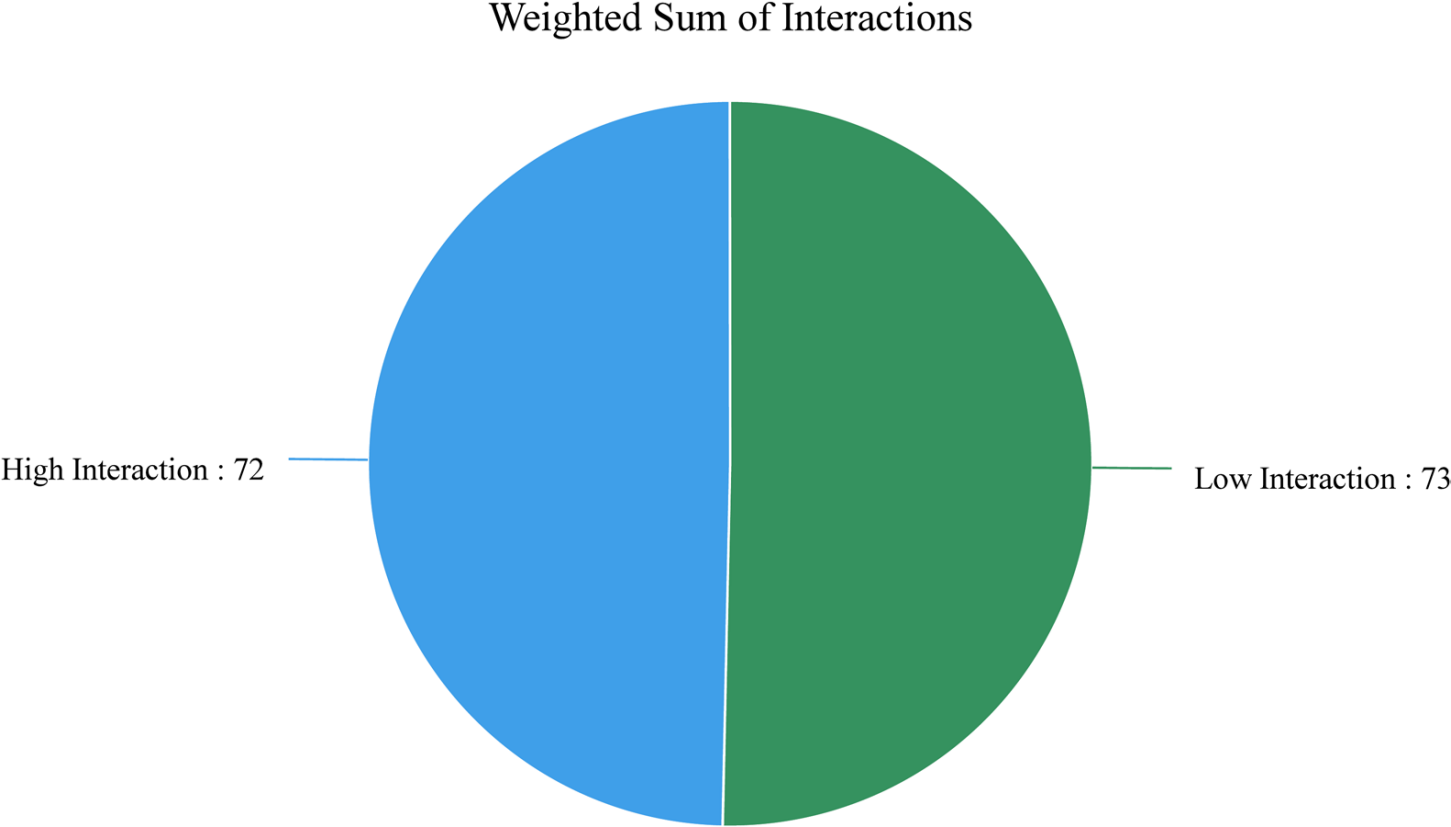
Interaction proportion.

The entropy weight method was used to objectively weight the four interaction indicators. Results showed that the number of saves had the highest weight (35.71%), followed by likes (32.86%) and comments (28.99%), while shares had the lowest weight (2.43%) (Supplementary Table 1). Based on the median comprehensive interaction score, videos were divided into a high-engagement group (n = 72) and a low-engagement group (n = 74) (Fig. 2).

**Fig. 3:**
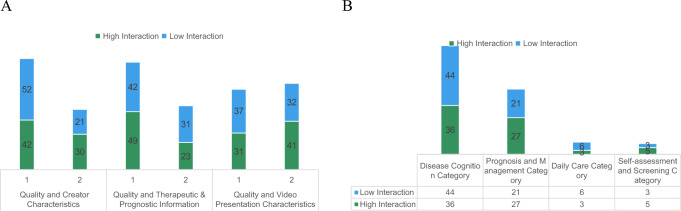
Distribution of interaction level association of short videos related to macular degeneration. (**A**) Distribution of high/low interaction based on clustering of different dimensions; (**B**) Distribution of high/low interaction of different theme categories. “High Interaction” in the figure represents the high-interaction group, “Low Interaction” represents the low-interaction group, and the height of the bar chart reflects the frequency or proportion of the corresponding group.

Characteristics of high-engagement videos included: regarding content theme, videos with "self-test and screening" themes had the highest proportion of high engagement (62.5%), followed by "prognosis and management" (56.25%), “disease cognition” (45.0%), and “daily care” had the lowest (33.3%) (Fig. 3). Regarding creator identity, although media accounts did not have the highest information quality scores, their median PEMAT-U score reached 0.714, significantly higher than other creator types, and they ranked first in interaction metrics: median likes 1187.00 (Q1 = 530.0, Q3 = 1844.0), median comments 32.00 (Q1 = 30.0, Q3 = 34.0), median saves 517.50 (Q1 = 70.0, Q3 = 965.0), and median shares 239.50 (Q1 = 161.0, Q3 = 318.0) (Supplementary Table 2). Interaction indicators showed extremely strong positive correlations: likes were significantly correlated with comments (r = 0.903, *P* = 0.000), saves (r = 0.949, *P* = 0.000), and shares (r = 0.892, *P* = 0.000). The number of followers was also significantly positively correlated with all interaction indicators (r = 0.334–0.476, *P* < 0.001) (Supplementary Table 3).

### Factors influencing information quality

Through cluster analysis, videos were divided into a high-quality group (cluster_1) and a low-quality group (cluster_2). The high-quality group had significantly higher scores in JAMA, mDISCERN, GQS, PEMAT-U, and PEMAT-A compared to the low-quality group (Table 2).

**Table 2:** Cluster analysis results of age-related macular degeneration short videos by key dimensions (n = 145).

Quality and creator characteristics								
	JAMA score	mDISCERN score	GQS score	PEMAT-U	PEMAT-A	Doctor’s professional title	Uploader	SC
Cluster_1 (64.83%)	0.172	0.39	0.498	0.391	0.557	3	4	0.307
Cluster_2 (35.17%)	− 0.317	− 0.718	− 0.919	− 0.721	− 1.026	3	4	

Quality and therapeutic and prognostic information								
	JAMA score	mDISCERN score	GQS score	PEMAT-U	PEMAT-A	Treatment	Prognosis	SC
Cluster_1 (62.76%)	0.186	0.397	0.519	0.415	0.583	1	0	0.301
Cluster_2 (37.24%)	− 0.314	− 0.669	− 0.874	− 0.7	− 0.982	1	1	

Quality and video presentation characteristics								
	JAMA score	mDISCERN score	GQS score	PEMAT-U	PEMAT-A	Video Format	Wearing White Coat	SC
Cluster_1 (64.83%)	0.172	0.39	0.498	0.391	0.557	2	2	0.307
Cluster_2 (35.17%)	− 0.317	− 0.718	− 0.919	− 0.721	− 1.026	2	2	

SC = silhouette coefficient.

Uploader:1 = Media, 2 = Physicians without ophthalmology background, 3 = Traditional Chinese Medicine Ophthalmologist, 4 = Western Medicine Ophthalmologist; Doctor’s professional title:1 = Associate Chief Physician, 2 = Attending Physician, 3 = Chief Physician, 4 = others; Treatment: 1 = mentions treatment; 0 = does not mention treatment; Prognosis:1 = mentions prognosis; 0 = does not mention prognosis; Video Format:1 = Dialogue, 2 = Monologue Narrative; Wearing White Coat:1 = No, 2 = Yes.

**Fig. 4:**
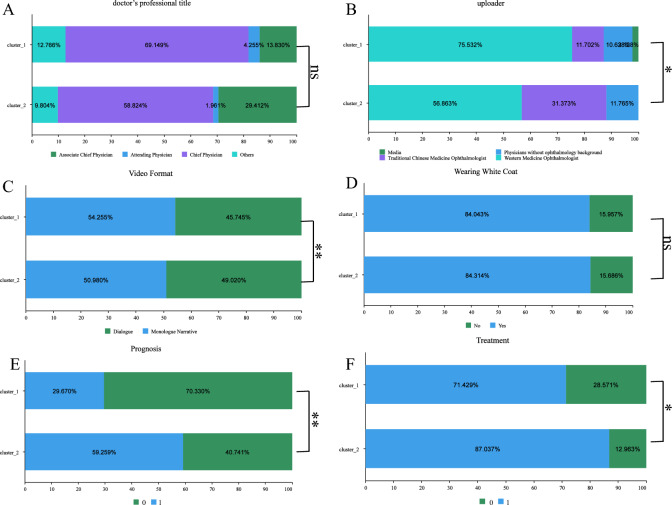
Multi-dimensional distribution characteristics of different clusters of short videos related to macular degeneration. (**A**) Distribution of doctors’ professional titles in different clusters; (**B**) Distribution of uploader types in different clusters; (**C**) Distribution of video formats in different clusters; (**D**) Distribution of whether wearing white coats in different clusters; (**E**) Distribution of prognosis mentions in different clusters; (**F**) Distribution of treatment mentions in different clusters ("*" indicates *P* < 0.05, "**" indicates *P* < 0.01, “ns” indicates*P* > 0.05).

Characteristics of high-quality videos included: creator identity predominantly Western medicine ophthalmologists (75.53%); content more likely to include prognostic information, with a mention rate of 70.33%; and video format predominantly monologue narration, accounting for 54.26%. Low-quality videos were more often created by Traditional Chinese Medicine (TCM) ophthalmologists, with the proportion of TCM ophthalmologists in the low-quality group rising to 31.37%, and they less frequently mentioned prognosis (mention rate 40.74%). Regarding video format, the low-quality group had a relatively higher proportion of dialogue format (49.02%), showing a significant difference from the high-quality group (*P* < 0.01) (Fig. 4).

Different uploader types showed no overall significant differences in JAMA, mDISCERN, and GQS scores (*P* > 0.05); however, significant overall differences were observed in PEMAT-U and PEMAT-A scores (*P* < 0.05). Media accounts had a PEMAT-U score of 0.714, higher than the other three creator types. Notably, physician professional title was not significantly associated with information quality scores (*P* > 0.05) (Supplementary Table 2).

### Form elements and content theme analysis

Video format (including dialogue and monologue narration) and whether the presenter wore a white coat had no significant impact on any quality scores, video duration, interaction indicators, or number of followers (*P* > 0.05) (Supplementary Table 3). However, in cluster analysis, differences in video format distribution were observed: the high-quality group had 54.26% monologue narration and 45.75% dialogue; the low-quality group had 49.02% dialogue and 50.98% monologue narration (*P* < 0.01) (Fig. 4). The proportion of presenters wearing white coats exceeded 84% in both groups, with no significant difference (Fig. 4).

**Fig. 5:**
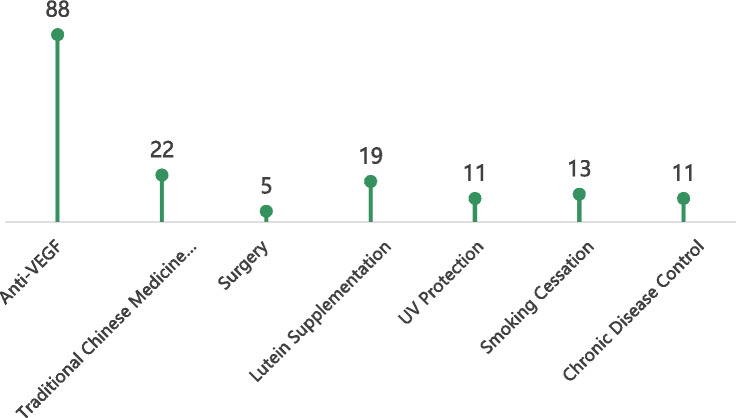
Summary of intervention measures: number of mentions of relevant intervention measures.

Different content themes showed marked differences in engagement levels (Fig. 3). The “disease cognition” category included 80 videos, with 45.0% high engagement; the "prognosis and management" category included 48 videos, with 56.25% high engagement; the “daily care” category had a small number of videos, with only 33.3% high engagement; the "self-test and screening" category had only 8 videos but achieved the highest proportion of high engagement at 62.5%. Regarding intervention measures, anti-vascular endothelial growth factor (Anti-VEGF) therapy was mentioned most frequently (88 times), followed by Traditional Chinese Medicine treatment (22 times), while surgical content was mentioned least (5 times). Lutein supplementation (19 times), smoking cessation (13 times), UV protection (11 times), and chronic disease control (11 times) were mentioned with similar frequency (Fig. 5).

## Discussion

With the rise of short video platforms, health education content has become an important channel for the public to obtain medical information. AMD is one of the leading eye diseases causing severe visual impairment and blindness in the global elderly population^20^. With the intensification of population aging, the prevalence and number of AMD patients continue to increase, which has become an increasingly serious public health problem^21^. Therefore, utilizing short videos for disseminating AMD health education content on public platforms is of great significance in enhancing public awareness of eye health, promoting early detection, and standardizing treatment. However, with the surge in information volume, problems such as the content quality and communication efficiency of short videos have become increasingly prominent, making it particularly critical to systematically evaluate and analyze ophthalmology health education content on short video platforms^22,23^. This study focused on health education content videos about AMD on the TikTok platform, using a series of internationally recognized assessment tools such as JAMA, mDISCERN, GQS, and PEMAT to conduct a systematic evaluation from multiple dimensions including information quality, comprehensibility, user interaction, and uploader characteristics, aiming to reveal the current status and problems of AMD health education content. Based on the statistical analysis results, the following will provide an in-depth discussion on video quality, communication effectiveness, and their influencing factors.

### Generally low information quality and urgent need for improved understandability

In this study, AMD health education videos generally received low scores on internationally recognized assessment tools such as JAMA, mDISCERN, and GQS. JAMA scores were concentrated at 1 point, indicating that the vast majority of videos severely lacked information traceability: although most provided author qualifications, they rarely indicated information sources, references, or conflicts of interest, making it difficult for users to discern the accuracy and authority of the information. mDISCERN and GQS scores were concentrated at 2–3 points, reflecting generally insufficient content depth, with most videos only covering superficial aspects of disease definitions and failing to include key information such as evidence levels and uncertainties in treatment options. The median PEMAT-U score was only 0.429, a noteworthy finding in this study. It is speculated that creators, accustomed to using professional ophthalmology terminology in their daily work, failed to effectively transform AMD-related knowledge into easily understandable expressions, resulting in limited transmission efficiency of health education content.

### Information quality and user engagement driven by different factors

The core finding of this study is that information quality and user engagement exhibit a significant but imperfect positive correlation. Correlation analysis revealed that various interaction indicators were significantly positively correlated with some quality scores (especially PEMAT-U and PEMAT-A) (r values 0.2–0.3, *P* < 0.05), and cluster analysis showed that over 70% of videos in the high-quality group achieved high engagement. However, approximately 30% of high-quality videos fell into the low-engagement group, while some low-quality videos achieved high engagement. This phenomenon suggests that although information quality and user engagement are interrelated, they are driven by different factors, providing important insights for understanding the dissemination patterns of health education content on short video platforms.

The drivers of information quality were primarily concentrated in creator identity and content depth. High-quality videos were predominantly created by Western medicine ophthalmologists (75.53%) and were more likely to include prognostic information (70.33%). This indicates that professional background and clinical relevance of content form the foundation for ensuring information accuracy and completeness. Notably, physician professional title was not significantly associated with information quality—chief physicians and attending physicians showed no differences in various quality scores, suggesting that professional title level does not necessarily translate into the professional depth of health education content.

In contrast, the drivers of user engagement were more multifaceted. First, the alignment between content theme and user needs was crucial: videos with "self-test and screening" themes had the highest proportion of high engagement (62.5%), while “daily care” had the lowest (33.3%). This difference reflects user preferences in health information seeking—compared to generalized daily health advice, users are more concerned with practical information that can be directly applied to their own risk assessment. Second, although media accounts did not excel in information quality scores, their median PEMAT-U score of 0.714 and various interaction metrics ranked highest, indicating their proficiency in transforming professional content into accessible language that precisely matches the information reception habits of platform users. The entropy weight method further showed that saves had the highest weight (35.71%), far exceeding shares (2.43%), indicating that users prefer to save videos for future reference rather than share them in public social circles.

### Mismatch between content theme distribution and clinical practice needs

In this study, anti-VEGF therapy was the most frequently mentioned intervention (88 times). As the current first-line treatment for wet AMD, its prominence in health education content aligns with clinical practice. Surgical content appeared only 5 times, possibly because this treatment is primarily used for specific complications of AMD. Notably, as a chronic fundus disease that is preventable and controllable but requires long-term management, current health education content remains highly concentrated on treatment aspects, with insufficient coverage of whole-process health management including risk factor prevention and control (such as smoking cessation, dietary adjustment, chronic disease management), early screening, and long-term follow-up. Videos on "self-test and screening" numbered only 8, and although they achieved the highest engagement proportion, their absolute number was extremely small, failing to meet users’ explicit needs. This mismatch between content theme distribution and clinical practice needs reflects limitations in the comprehensiveness and systematicity of current health education content systems.

### Limited impact of form elements and implications for creator strategies

This study found that video format (dialogue vs. monologue narration) and whether the presenter wore a white coat had no significant impact on any quality scores or interaction indicators. This negative finding has important practical implications: creators need not overly focus on superficial formal packaging and should instead prioritize content accuracy, practicality, and understandability. However, cluster analysis revealed differences in video format distribution between groups (the high-quality group had slightly more monologue narration, while the low-quality group had slightly more dialogue). Since direct comparisons showed no significant differences in quality scores between the two formats, this distribution difference further supports the limited role of formal elements.

Based on the above findings, future AMD health education content creation could be optimized in the following aspects: for medical professionals, efforts should focus on improving content understandability, actively transforming professional terminology into accessible language, and adding user-concerning content such as prognostic information; for media accounts, while maintaining their communication technique advantages, they should strengthen information source attribution and content depth to enhance information credibility and completeness. This suggests that the effective combination of professional content and communication skills may be an important pathway to improving the quality and dissemination effectiveness of health education videos.

## Research limitations and prospects

The data source of this study was limited to the single platform of TikTok. Future studies can include more short video platforms for comparison to enhance the generalizability of the research conclusions. Secondly, this study adopted a cross-sectional design, which has issues such as insufficient data timeliness and difficulty in establishing causal relationships between variables. Subsequent research could be extended by conducting multiple data collections at different time points, combining longitudinal tracking, or expanding the sample scope. Furthermore, although the quality assessment of video content used internationally common tools, there are still limitations of subjective judgment. Future studies could introduce objective methods such as user cognitive experiments as supplements.

## Conclusion

This study demonstrates that AMD health education videos on the TikTok platform generally suffer from poor information quality, with information quality and user engagement driven by different factors: information quality primarily depends on creators’ professional background and the completeness of health education content, while user engagement is more influenced by the alignment between content themes and user needs, as well as content understandability. It is recommended that future health education creation, while ensuring professional accuracy, strengthen information source attribution, improve content understandability and practicality, and promote thematic diversification. Platform operators may consider incorporating save rates and quality scores into recommendation algorithms to optimize traffic distribution mechanisms and promote the effective dissemination of high-quality health education content.

## Supplementary Information

Supplementary Information 1.

Supplementary Information 2.

## Data Availability

The datasets generated during and/or analysed during the current study are available from the corresponding author on reasonable request.
