# Supplementary material for: Assessment of quality, reliability, and interactive characteristics of age-related macular degeneration short videos on TikTok: a cross-sectional study
Source: Sci Rep. 2026 Apr 30;16:20047. doi: 10.1038/s41598-026-44509-1 (PMC13319755; doi:10.1038/s41598-026-44509-1)
Supplement: Supplementary file 1 — Supplementary Information 1. [file 41598_2026_44509_MOESM1_ESM.pdf]

Supplementary Materials

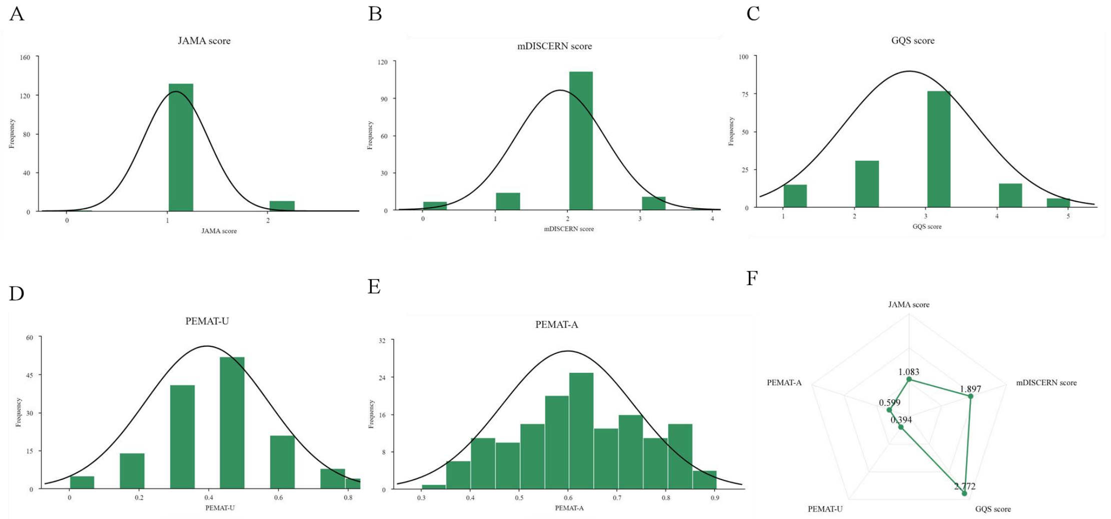

Supplementary Figure 1 Frequency distribution of short video quality scoring indicators related to macular degeneration

A: JAMA score frequency distribution;

B: Frequency distribution of mDISCERN scoring;

C: Frequency distribution of GQS scores;

D: PEMAT-U frequency score;

E: Frequency distribution of PEMAT-A;

F: Radar chart:Comprehensive visualization compares the scoring performance of multiple quality assessment indicators, including JAMA, mDISCERN, GQS, PEMAT-U, and PEMAT-A.

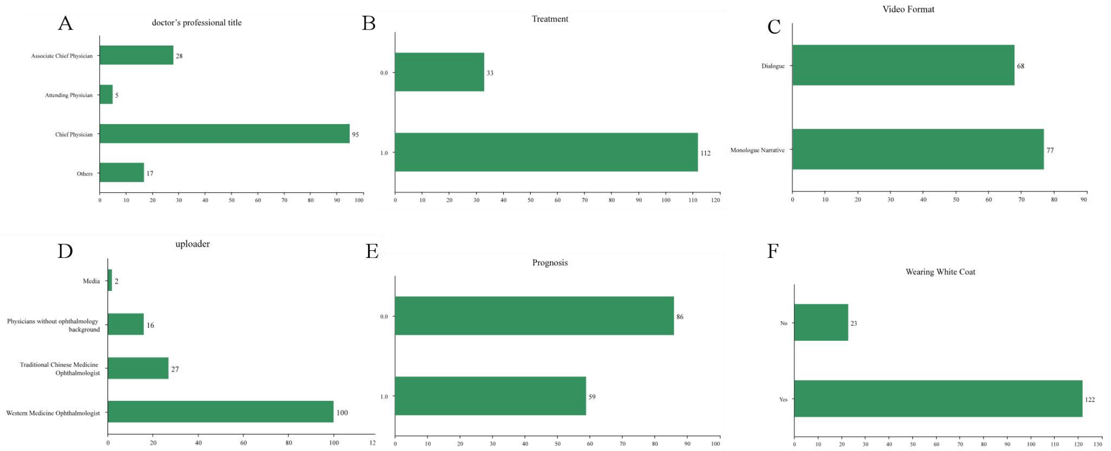

Supplementary Figure 2 Frequency Distribution of Quality Score Indicators of Short Videos Related to Macular Degeneration

A: Doctor's professional title frequency distribution;

B: Treatment mention frequency distribution;

C: Video format frequency distribution;

D: Uploader type frequency distribution;

E: Prognosis mention frequency distribution;

F: Frequency distribution of whether wearing white coat

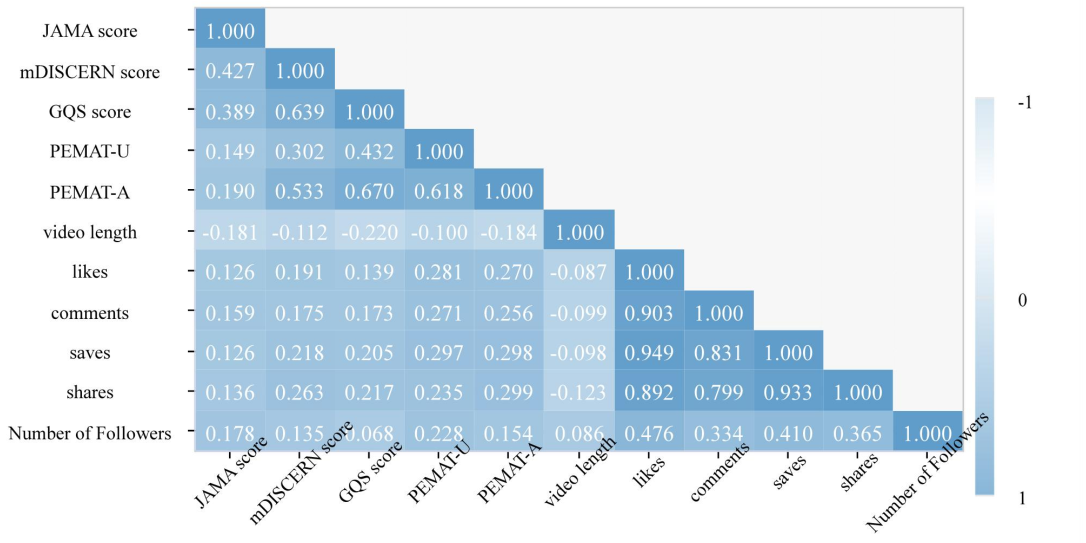

Supplementary Figure 3 Spearman Correlation Analysis of Core Indicators of Short Videos Related to Macular Degeneration

Supplementary Table 1 Weight Coefficients of Short Video Interaction Indicators Calculated by Entropy Weight Method (n=145)

Indicator	Entropy	Information utility value	weight coefficient
**likes**	0.9907	0.0093	9.66%
**comments**	0.9668	0.0332	34.52%
**saves**	0.9787	0.0213	22.18%
**shares**	0.9677	0.0323	33.64%

Note:lin-* = Logarithmically normalized indicator; Entropy = Information entropy; Information utility value = 1 - Entropy.

Supplementary Table 2-1：Non-Parametric Test Results of Core Indicators by Uploader Type (n=145)

Indicator	Media	Physicians without ophthalmology background	Traditional Chinese Medicine Ophthalmologist	Western Medicine Ophthalmologist	P
**JAMA score**	1(1.0,1.0)	1.000(1.0,1.8)	1 (1.0,1.0)	1.000(1.0,1.0)	0.086
**mDISCERN score**	2(2.0,2.0)	2.000(2.0,2.0)	2.000(1.0,2.0)	2.000(2.0,2.0)	0.281
**GQS score**	3.000(2.0,4.0)	3.000(2.3,3.8)	2.000(2.0,3.0)	3.000(2.0,3.0)	0.055
**PEMAT-U**	0.714(0.6,0.9)	0.286(0.1,0.4)	0.286(0.3,0.4)	0.429(0.3,0.6)	0.002
**PEMAT-A**	0.775(0.8,0.8)	0.600(0.5,0.7)	0.550(0.5,0.6)	0.600(0.5,0.7)	0.005
**video length**	1.000(1.0,1.0)	1.000(1.0,1.8)	2.000(1.0,2.0)	1.000(1.0,2.0)	0.006
**likes**	1187.000(530.0,1844.0)	39.000(13.3,118.5)	115.000(52.0,283.0)	233.000(72.8,846.8)	0.000
**comments**	32.000(30.0,34.0)	2.500(0.3,5.0)	5.000(3.0,16.0)	13.500(5.0,64.8)	0.000
**saves**	517.500(70.0,965.0)	8.000(2.5,46.3)	32.000(16.0,187.0)	78.500(18.0,429.0)	0.002
**shares**	239.500(161.0,318.0)	14.000(3.0,35.8)	15.000(7.0,115.0)	42.000(11.3,260.5)	0.049
**Number of Followers**	251.950(82.2,421.7)	4.200(0.6,42.5)	7.200(1.6,26.5)	2.500(0.9,8.9)	0.033

Note:Data are presented as median (Q1, Q3); Statistical analysis: Kruskal-Wallis H test; *P*<0.05 was considered statistically significant; TCM = Traditional Chinese Medicine.

Supplementary Table 2-2：Non-Parametric Test Results of Core Indicators by Physician’s Professional Title(n=145)

Indicator	Associate Chief Physician	Attending Physician	Chief Physician	Others	P
**JAMA score**	1.000(1.0,1.0)	1.000(1.0,1.0)	1.000(1.0,1.0)	1.000(1.0,1.5)	0.065
**mDISCERN score**	2.000(2.0,2.0)	2.000(1.5,2.5)	2.000(2.0,2.0)	2.000(2.0,2.0)	0.56
**GQS score**	3.000(2.0,3.0)	3.000(2.0,3.5)	3.000(2.0,3.0)	3.000(2.5,4.0)	0.4
**PEMAT-U**	0.429(0.3,0.5)	0.429(0.4,0.6)	0.429(0.3,0.4)	0.286(0.1,0.6)	0.478

**PEMAT-A**	0.525(0.5,0.7)	0.600(0.5,0.8)	0.600(0.5,0.7)	0.600(0.6,0.7)	0.591
**video length**	1.000(1.0,2.0)	2.000(1.0,2.0)	1.000(1.0,2.0)	1.000(1.0,1.5)	0.471
**likes**	150.000(45.3,292.0)	61.000(13.0,1090.0)	214.000(79.0,792.0)	41.000(15.5,136.0)	0.001
**comments**	7.000(2.3,30.8)	5.000(2.0,29.0)	11.000(5.0,52.0)	3.000(0.5,5.5)	0.001
**saves**	35.000(16.0,141.5)	38.000(5.5,939.5)	75.000(18.0,342.0)	8.000(3.0,59.5)	0.018
**shares**	20.500(5.3,96.8)	89.000(2.5,656.5)	42.000(12.0,228.0)	18.000(4.0,55.5)	0.229
**Number of Followers**	2.900(0.4,9.8)	0.220(0.1,1.2)	2.900(0.9,10.4)	5.600(0.7,56.8)	0.039

Note: Data are presented as median (Q1, Q3); Statistical analysis: Kruskal-Wallis H test; $P<0.05$ was considered statistically significant; TCM = Traditional Chinese Medicine.

Supplementary Table 3-1 Non-Parametric Test Results of Core Indicators by Video Format (n=145)

Indicator	Dialogue	Monologue Narrative	*P*
**JAMA score**	1(1.0,1.0)	1(1.0,1.0)	0.507
**mDISCERN score**	2(2.0,2.0)	2(2.0,2.0)	0.533
**GQS score**	3(2.0,3.0)	3(2.0,3.0)	0.948
**PEMAT-U**	0.429 (0.3,0.4)	0.429 (0.3,0.4)	0.789
**PEMAT-A**	0.6 (0.5,0.7)	0.6 (0.5,0.7)	0.622
**video length**	1(1.0,2.0)	1(1.0,2.0)	0.782
**likes**	118.5 (55.0,334.3)	214 (61.5,890.5)	0.091
**comments**	7.5 (3.0,23.8)	11 (4.5,52.5)	0.060
**saves**	38 (9.3,169.0)	70 (18.5,344.0)	0.093
**shares**	22.5 (5.0,124.0)	42 (12.5,235.0)	0.069
**Number of Followers**	2.35 (0.5,6.9)	5 (1.0,17.9)	0.069

Note: Data are presented as median (Q1, Q3); Statistical analysis: Mann-Whitney U test; $P<0.05$ was considered statistically significant.

Supplementary Table 3-2 Non-Parametric Test Results of Core Indicators by Presenter Attire (n=145)

Indicator	No white coat	White coat	P
**JAMA score**	1.000(1.0,1.0)	1.000(1.0,1.0)	0.806
**mDISCERN score**	2.000(2.0,2.0)	2.000(2.0,2.0)	0.903
**GQS score**	3.000(2.0,3.0)	3.000(2.0,3.0)	0.607
**PEMAT-U**	0.429(0.3,0.6)	0.429(0.3,0.4)	0.217
**PEMAT-A**	0.550(0.5,0.8)	0.600(0.5,0.7)	0.853
**video length**	1.000(1.0,2.0)	1.000(1.0,2.0)	0.126
**likes**	115.000(44.0,520.0)	191.000(60.8,513.0)	0.583
**comments**	7.000(3.0,27.0)	9.000(4.0,34.0)	0.409
**saves**	29.000(8.0,174.0)	57.000(15.0,244.3)	0.359
**shares**	16.000(5.0,88.0)	35.500(8.0,224.3)	0.277
**Number of Followers**	5.900(0.4,17.9)	2.700(0.9,9.8)	0.667

Note: Data are presented as median (Q1, Q3); Statistical analysis: Mann-Whitney U test; $P<0.05$ was considered statistically significant.
